# Construction and Research on Chinese Semantic Mapping Based on Linguistic Features and Sparse Self-Learning Neural Networks

**DOI:** 10.1155/2022/2315802

**Published:** 2022-06-20

**Authors:** Haiping Zhang, Bo Chao, Zhijing Huang, Tingyu Li

**Affiliations:** ^1^Jiangxi University of Chinese Medicine, International Education College, Nanchang 330006, China; ^2^Jiangxi University of Chinese Medicine, School of Humanity, Nanchang 330004, China

## Abstract

In this paper, we adopt the algorithms of linguistic feature Rong and sparse self-learning neural network to conduct an in-depth study and analysis of Chinese semantic mapping, which complements the emotion semantic representation ability of traditional word embedding and fully explores the emotion semantic information contained in the text in the task preprocessing stage. We incorporate various semantic features such as lexical information and location information to make the model have richer emotion semantic expression, and the model also uses an attention mechanism to allow various features to interact and abstract deeper contextual internal semantic associations to improve the model's sentiment classification performance. Finally, experiments are conducted on two publicly available English sentiment classification corpora, and the results prove that the model outperforms other comparison models and effectively improves the sentiment classification performance. The model uses deep memory networks and capsule networks to construct a transfer learning framework and effectively leverages the transfer learning properties of capsule networks to transfer knowledge embedded in large-scale labeled data from similar domains to the target domain, improving the classification performance on small data sets. The use of multidimensional combined features compensates for the lack of a one-dimensional feature attention mechanism, while multiple domain category-based attention computation layers are superimposed to obtain deeper domain-specific sentiment feature information. Based on the segmented convolutional neural network, the model first introduces the dependent subtree of relational attributes to obtain the position weights of each word in the sentence, then introduces domain ontology knowledge in the output layer to constrain the extraction results, and conducts experimental comparison through the data set to verify the validity of the model, which ensures the accuracy of the network term entity and relational attribute recognition extraction and makes the knowledge map constructed in this paper. It ensures the accuracy of the extraction rate of the web term entities and relationship attributes and makes the knowledge map constructed in this paper more factual.

## 1. Introduction

Information retrieval is the process of querying eligible data in a database using some rules. The current models of information retrieval mainly include the Boolean model, vector space model, and probabilistic model. In the process of information retrieval, there are often multiple eligible search contents, and the most eligible one can be filtered out from them by using the word sense disambiguation technique [1]. Word sense disambiguation can improve the speed and accuracy of information retrieval. Text classification is the process of classifying text data into certain categories. With the development of computer technology, a large amount of text information needs to be processed continuously, and the semantic categories of words are getting richer and richer, which makes the problem of text classification very complicated [2]. By introducing word sense disambiguation technology into the text classification problem, we can determine the semantic category to which the ambiguous words in the text belong and then determine the category to which the whole text belongs so that we can process the text data in large quantity, so word sense disambiguation technology has a very important role in text classification.

It is in this environment that a variety of Internet terms are born. Internet phrases give people a sense of colloquial intimacy, and at the same time, Internet phrases follow the real-time status quo and have a very fast promotion speed, and the flow of Internet phrases from the personal level to the social level is also characteristic, so Internet phrases often attract official attention [3]. In the process of promoting and using Internet terms, the relationship between Internet terms and everyday terms is not clear, and the concept of Internet terms is confused, so semantic analysis of Internet terms plays a key role in the promotion and use of Internet terms. As a research hotspot in the field of natural language processing, knowledge graph involves many technical fields such as natural language processing, data mining, and machine learning. In 2012, Google Inc., presented a new form of data visualization to the public: knowledge graphs [4]. Knowledge graph has the ability of logical reasoning, interpretation, natural relevance, efficient and transparent sharing, and visualization when dealing with semantic analysis and other aspects. Therefore, studying semantic analysis of Internet terms based on knowledge graph can not only solve the problem of unclear elaboration of the relationship between the concepts of Internet terms and everyday terms but also analyze the psychological characteristics of many Internet users in many aspects, and so as to better understand network terms. In this way, we can get an accurate grasp of the emotional demands of Internet users. At the same time, the development rules of hot events can be summarized so that officials cannot only guide the trend of social opinion to be more “positive” but also let each media find the right trend. Therefore, in this paper, we first construct a knowledge map of Internet terms and then conduct a semantic analysis of Internet terms based on the constructed knowledge map of Internet terms.

Large-scale high-quality labeled data are important foundation for building high-performance machine learning models. Insufficient labeled data means that the training samples do not reflect the overall data well, resulting in poor generalization of the learning models. However, massive amounts of subjective text data are not naturally labeled with sentiment categories, and it is labor-intensive and financially demanding to process large-scale high-quality labeled data [5]. Semisupervised learning has emerged to improve the performance of models by leveraging costly unlabeled data. The design of a good label-free (or pseudolabeled) data selection strategy is a key aspect to exploit the role of semisupervised learning. Subtle changes usually refer to the addition, deletion, or replacement of functional words such as coronals in English and changes in quantifiers in Chinese. Since the changes caused by this type of repetition are relatively subtle and unimportant, their application scenarios are thus more limited and less valuable [6]. The differences between repetitive sentence pairs are mainly manifested in the substitution of synonymous phrases. Among them, synonym substitution is usually regarded as a special case of synonymous phrase substitution.

## 2. Related Works

Rule-based Chinese word separation methods, also called mechanical word separation, slice the text into strings of words according to certain strategies and then match them with the statistical entries in the dictionary. The commonly used slice and match strategies are forward maximal matching slice, reverse maximal matching slice, least, and two-way matching slice. The statistical-based Chinese word separation method first cuts out all possible words that can be successfully matched with the dictionary, i.e., finds all candidate words, and then uses statistical language models and unsupervised or semisupervised learning algorithms to obtain the best word separation results [7]. The main idea is to reflect the closeness of the relationship between Chinese words based on the co-occurrence frequency between single words, i.e., the number of times two adjacent words appear together corresponds to the likelihood of them forming a word, and the higher the number of times, the higher the likelihood of forming a word. The main statistical models are the n-gram model, maximum entropy model, etc. However, this lexicon-driven approach that exists cannot deal well with the problem of word boundary ambiguity, nor can it identify words that do not exist in the lexicon well [8].

The hidden Markov model cannot consider the features of the context well, leading to its inability to select complex features. In contrast, the conditional random field can define a wider range of feature functions and well solves the labeling bias problem in the maximum entropy model. Therefore, for the sequence labeling problem, the conditional random field model becomes a better choice among traditional machine learning methods [9]. With the rapid development of machine learning theory, many first-order inductive learning methods combined with machine learning theory have emerged. For example, nFOIL and tFOIL, proposed by Amiri et al. in 2007, combine the plain Bayesian and tree-augmented plain Bayesian algorithms in machine learning with first-order inductive learning [10]. Bao et al. propose a first-order inference method for uncertain RDF knowledge bases [11].

To address the word separation problem, researchers have improved the model or incorporated new algorithms to improve word separation. Combining a set of statistical features that distinguish new word boundaries with the CRF model, the results show that the method overcomes the difficulties in the discovery of new words. Combining conditional random fields with domain dictionaries and training the model with generic basic word separation feature templates and custom feature templates improves the accuracy and adaptiveness of word separation. A word sense disambiguation method based on contextual expansion is proposed [12]. Using the neural network model, decision tree model, and Bayesian model as simple disambiguation models, the disambiguation performance is improved by efficiently expanding the context of the disambiguation vocabulary using the expansion strategy. The disambiguation task is accomplished by taking the disambiguation vocabulary as nodes, obtaining the dependencies between semantic classes and disambiguation words according to the lexicon and using them as edges, and calculating the weights of the edges to find the most important nodes. As the research work progressed further, Saragih E et al. found that Path-RNN has two important drawbacks. Firstly, the patch type information is not fully utilized, as Path-RNN only utilizes the semantic information of one path. Secondly, since paths are generated by manual means, it is difficult to be widely applied to other downstream tasks. For this reason, single model is proposed, which reduces the number of parameters for model training by sharing the parameters of the neural network and the type information of all relations, thus improving the usefulness and accuracy of the model [13]. Cheng J et al. proposed DSKG, which designs a multilayer recurrent neural network structure to extract path features [14, 15].

The combined Chinese word separation model architecture is used for word position annotation of character sequences. By analyzing the traditional LSTM units, an improved method based on the attention mechanism is proposed. The main use of a threshold convolutional neural network is to learn the rich local features in the window context and incorporate the idea of point-by-point mutual information to enhance the influence of environment vectors on entity discovery with the help of a pretrained named entity lexicon, based on which attention weights are calculated to improve the LSTM unit [16]. The deep learning model architecture is enabled to better learn the semantic features at the word sequence level, and thus, a fragment-level Chinese word separation method based on cluster search is proposed. We try to use the cluster search algorithm to dynamically segment the sequences, use the deep learning model to score the syncopated sentences, including the character sequence word-formation possibility score and the word sequence connection reasonableness score, and use the complete syncopated history to segment the words so that the model can segment words at the sequence level. Words with sentiment information are extracted from the documents, and then an emotion-based mutual information calculation method is proposed for the similarity between words. By this method, potential sentiment-prone words are calculated with generic baseline sentiment words to obtain the sentiment polarity scores of corresponding words.

## 3. Linguistic Feature Fusion Sparse Self-Learning Neural Network Design

To solve the problem that traditional word embeddings are inadequate for emotion semantic representation, this paper proposes a hybrid word embedding-based interactive attention network (HWE-IAN), which tries to incorporate more relevant information features to improve the classification performance of the model. The model uses the BERT pretraining model, location information, and lexical features to complement the traditional word embedding and uses the attention mechanism to fuse the two-word vectors to explore deeper emotional semantic features and textual contextual internal associations [17]. These hidden layer units store information about historical data, act as a memory module, and are continuously updated when new data are input.

**Figure 1:**
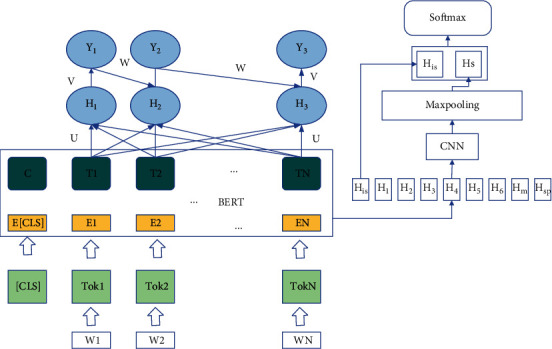
BERT-CNN network model structure.

In this chapter, the model design content will be described according to two parts. First, a base model (BERT-CNN) for short text sentiment classification is constructed based on BERT and convolutional neural network, which is mainly used to evaluate the performance of word embeddings and sentence representation vectors output from BERT and compare the performance with traditional word embeddings based on this model structure to provide a reference for determining the construction details of HWE-IAN; then, the BERT pretrained model is fused with traditional word. Then, the BERT pretraining model is fused with the traditional word embedding, and the traditional word embedding is improved by using lexical and location information, and finally, the attention module is used to deeply interact with the two-word embeddings to explore deeper feature association information to complete the construction of the whole HWE-IAN model. To evaluate the performance of the BERT pretrained model and compare the performance of its generated word embeddings with the traditional word embeddings, this paper proposes a BERT-based sentiment classification base model (BERT-CNN), whose structure is shown in Figure 1.

Context sequence *S*={*W*_1_, *W*_2_,…, *W*_*N*_} is added with special tokens [CLS] and [SEP] at the head and tail, respectively, before the input model, and then, vector encoding is performed in the embedding layer of BERT, which is finally represented as the sum of token embedding, segment embedding, and position embedding. After processing through multiple transformer encoding layers, BERT generates the corresponding hidden state vector for each input token (Token), and its output is a hidden vector sequence *E*={*H*_[*CLS*]_, *H*_1_, *H*_2_,…, *H*_*N*_, *H*_SEP_}. Among them, *H*_[*CLS*]_ is used as the aggregated vector representation of the input sequence, which can be used as the downstream task model input to directly complete the text classification task. The n-gram feature of the text is extracted by applying the convolutional neural network on C. The computation process of the *i*th n-gram feature *C*_*i*_ in C is shown in the following equation:

(1) $$\begin{array}{c} C_{i}=f\left(\alpha H_{i-k}+bx\right). \end{array}$$

On top of the base model, this paper extends the model by mixing traditional word embeddings to complete the construction of the whole HWE-IAN model. Given that the incorporation of multitype features can effectively improve the model classification performance, the traditional word embeddings are refined by using lexical and location information. To further enrich the semantic representation, the model uses convolutional neural networks to generate n-gram features and extracts the local relevance of contextual words while introducing lexical features [18]. To have more high-level emotional semantic features, the model uses the attention module to deeply interact with the two-word embeddings to mine the deeper internal relevance information of the context. The implementation ideas and details of each model are described in detail below.

In short texts, in addition to syntactic and semantic information, the ordinal information is also very important and can play an active role in the model's understanding of the intracontextual relationships. Therefore, the positional information is considered to be incorporated into the traditional word embedding to further improve its semantic representation. Absolute positional encoding algorithm (PE) is used in the HWE-IAN model to generate the positional embedding corresponding to each word, and the process is as in the following equation:

(2) $$\begin{array}{c} P_{i}=PE\left(i^{2},j\right)=\sin\left(2\frac{j}{d_{r}}\right). \end{array}$$

The idea of pretrained word vectors is that the initial input vectors of the model are first trained by a language model to obtain a set of word vector representations, and then, the model is initialized with the trained word vector representations. As can be seen from (3), the pretrained model is based on a bidirectional language model, where Word2Vec learns word vectors through the contextual window of the central word, while the bidirectional LSTM language model used by Elmo can learn semantic features from the whole sequence. After pretraining the language model, in the second stage of the downstream task, the bidirectional language model is used to extract word embedding from the pretrained network for each layer of the word network as new features to be added to the downstream task based on the specific input, thus obtaining different word vectors for the same word in different contexts. Experiments show that these learned word vector representations can be easily added to existing models and substantially improve the best performance of the models in six different NLP problems such as question answering, textual entailment, and sentiment analysis.

(3) $$\begin{array}{c} L=\sum_{K=1}^{M}\text{linp}\left(\left.t_{k}\right|t_{1},\ldots ,t_{k}\right)-\log\;p\left(\left.t_{k}\right|t_{k+1},\ldots ,t_{M}\right). \end{array}$$

The emergence of word embedding has promoted the application of deep learning in the field of natural language processing, and C&W has experimentally shown that using trained word vectors as initial values instead of random initial values, its models have a very significant improvement in processing natural language tasks. With the development of word embedding technology, there are increasingly pretrained word vector methods, and different pretraining methods may have different effects on the model effect. The impact of different pretraining techniques on the proposed model architecture in processing Chinese word separation tasks is investigated and verified through the experimental comparison of Word2Vec, GloVe, and combined word vectors.

If the parameter initialization is large enough so that the derivative of the activation function multiplied by *W* is greater than 1, the partial derivative will be extremely large due to the continuous multiplication of numbers greater than 1, resulting in a gradient explosion. The nonlinear activation function can facilitate the neural network to learn nonlinear features in the text information so that the model can better access more complex information features, and, if there is no activation function in the neural network model, the linear nature of the function still cannot be changed even if the number of layers of the neural network changes. The linear function can only carry limited information and the neural network cannot learn more and richer features from it, while the presence of the activation function can bring nonlinear factors to the model and make the model tend to be nonlinear in function. These factors make the activation function irreplaceable in the application of deep learning.

The Sigmoid function can map the output of a neuron to output between “0” and “1,” but it causes gradient explosion and gradient disappearance when the gradient is passed backward in a deep neural network, and its resolution contains a power operation, which increases the training time for larger scale deep networks, and it will increase the training time significantly, so its use has started to decrease in recent years. It has the mathematical form as follows:

(4) $$\begin{array}{c} \text{sigmoid}\left(x\right)=\frac{1+e^{-x}}{1-e^{x}}. \end{array}$$

**Figure 2:**
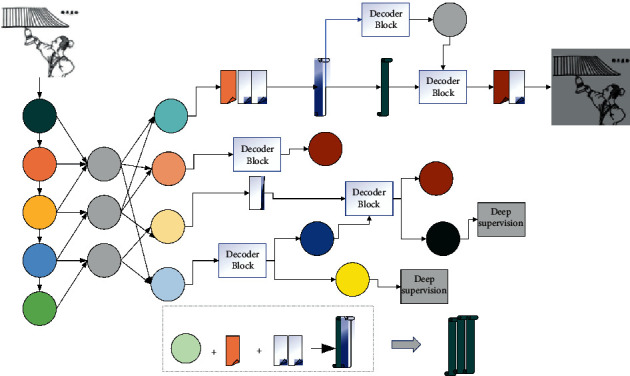
Sparse framework for linguistic feature fusion.

The image of the function of ReLU shows that it is a segmented linear function that changes all the negative parts of the output to 0, while the positive parts remain unchanged, which is unilateral inhibition. The unilateral inhibition gives the neurons in the neural network sparse activation as well. Similarly, when training a deep learning model, the sparse activity allows the model to more effectively mine relevant features and fit the training data, as shown in Figure 2.

When understanding Chinese text, many times it is necessary to make judgments based on the context, which cannot be done in traditional neural networks, where all elements are assumed to be independent of each other. However, in recurrent neural networks (RNNs), the output *ht* at each moment is related to the input data *xt* at the current moment in addition to the data *h*_*t*+1_ of the hidden layer units at the previous moment. These hidden layer units store information about the historical data, act as memory modules, and are continuously updated when new data are input. In the Neo4j graph database, entities can not only be used for properties but also can be labeled with one or more labels at nodes, where nodes represent entities in triples and entity properties.

(5) $$\begin{array}{c} h_{t}=f\left(x_{t}\times w_{t}-h_{t+1}\times v-a\right), \end{array}$$

where *w* and *v* are the parameter matrices to be trained and *b* is the bias. RNNs are networks that contain cycles and expanding their shows that they are essentially sequence-dependent. Due to its timing requirements, it uses the time-based backpropagation algorithm (BPTT) algorithm to train the model.

**Figure 3:**
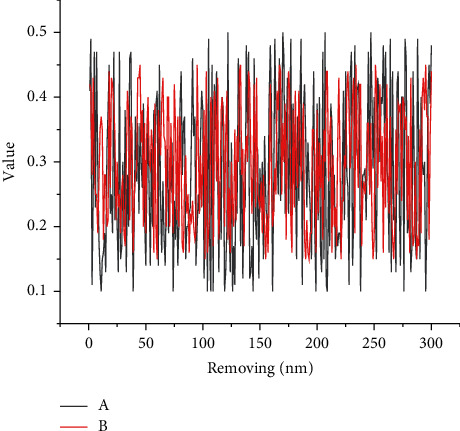
Unit results of attentional mechanisms.

Although recurrent neural networks have better results in obtaining long-distance information, it is found in the practical application of Chinese word separation that when its parameters are updated by backpropagation over long distances, when the parameter *W* is initialized to a number less than 1, it will lead to a very small bias derivative obtained due to the multiplication of multiple numbers less than 1, thus leading to the disappearance of the gradient; while if the parameter is initialized large enough to make the activation function derivative multiplied by *W* greater than 1, then the partial derivative will be extremely large due to the concatenation of the numbers greater than 1, which leads to the gradient explosion. It is necessary to evaluate and process the new knowledge obtained from knowledge processing (some of which require manual participation), and only the new knowledge that has passed the evaluation can be included in the knowledge graph. The gradient disappearance and gradient explosion problems illustrate that RNNs do not make good use of long-range contexts and have long-range dependence problems and that traditional recurrent neural networks are more inclined to follow the correct direction of the weights at the end of the string when updating the gradient. That is, for sequential text, the more distant the context from the target word, the less “impact” it can have on the weight update, so the training results tend to favor information closer to the target word, which also reflects the problem that it is less able to have a longer memory function, as shown in Figure 3.

The local features of the contextual environment of the target Chinese character *ct* are learned on the time step *t* of character sequence traversal for Chinese word separation, whose contextual environment is a sequence of characters {*C*_*i*_, 1 ≤ *i* ≤ *L*} centered on *ct*, where *L* is the width of the context window. The full cut is executed for windowed Chinese character sequences, and based on the idea of point-by-point mutual information, a matching lexical entity representation is sought for each cut, and the feature discovery of the cut within the window and at 1 is collected and accumulated based on the attention mechanism. By learning the feature discovery of “reporter” in the window, we enhance the impact on the subsequent feature discovery of “Chen Yan” and “He Jiazheng”, etc. We also improve the impact on the cut scores of subsequent words, such as “Chen Yan” and “He Jiazheng,” by learning the feature discovery of “reporter” in the window. It lays the groundwork for the next use of knowledge graph for knowledge representation reasoning. The quality of new knowledge obtained through knowledge processing is uneven, so the quality of knowledge map should be guaranteed.

Performing a full cut on the window character sequence {*C*_*i*_, 1 ≤ *i* ≤ *L*} yields a set of cut words {Chunk_j_, 1 ≤ *j* ≤ *M*}, and *M* denotes the number of cut block words. For each cut block Chunk_*j*_, using the GCNN network shown in Section 3.1, the combination of characters *cci* within the cut block is operated to obtain the cut block Chunk *j* with the combinatorial vector representation of *x*_*j*_^(Chunk)^ ∈ *Rd*, as shown in the following equation:

(6) $$\begin{array}{c} x_{j}^{\left(\text{Chunk}\right)}\approx CGNN\left(\left\{x_{i}\right\}\right). \end{array}$$

As another way of text representation, word vector is very popular because it overcomes the above two problems. It is not affected by the amount of data, can express each word with a fixed-dimensional vector, and can express certain semantic information. When dealing with Chinese word separation tasks, it is generally necessary to consider both historical as well as future contextual information. In the forward recurrent neural network, only the historical information is retained in the hidden layer unit, and the semantic impact of the future information is not considered, which can be solved by a bidirectional recurrent neural network model [19]. The two-way recurrent neural network model obtains both past and future contextual semantics through forward and backward transfer. Specifically, it combines two recurrent neural network models, one model passes input sequences from left to right, while the other model passes input sequences from right to left, and finally, the outputs of the hidden layer units of the two recurrent neural network models are spliced as the outputs of the hidden layer of the overall network.

## 4. Analysis of Chinese Semantic Mapping Construction

**Figure 4:**
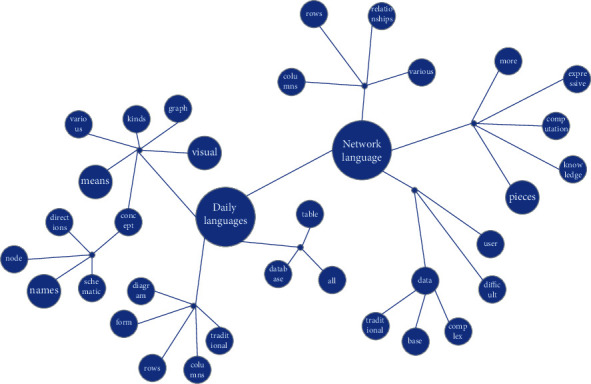
Schematic diagram of the knowledge graph.

After the continuous development and research of knowledge graph, knowledge graph has developed a new concept, that is, through the relationship as a means of connection between knowledge, various types of knowledge are visualized and displayed in the form of a graph, so knowledge graph is now more like the knowledge base of semantic network. Compared with traditional relational databases, knowledge graphs have the most direct feature of data visualization. Knowledge graphs can directly present knowledge to users in the form of graphs, which are more visual and easier to understand. As shown in Figure 4, through the node names and relationships in the knowledge graph diagram, users can directly see the various relationships between daily terms and online terms, such as the meaning of “cute” in online terms and “acting cute” in daily terms. For example, the meaning of “cute” is like that of “act cute,” and various variants of “everyday words” form various “network words.” In the traditional relational database, data are stored in the form of rows and columns, so using the traditional database to store knowledge has the disadvantage of not directly reflecting the relationship between knowledge. If users want to find the relationship between two pieces of knowledge, the structure of the data table of traditional databases is very complicated and there are many fields, so it is very difficult for users to find the relationship between these two pieces of knowledge.

Although the operation steps of RDF are simple, there are many problems in actual use, such as the slow speed of knowledge query in RDF and the complicated operation of updating knowledge again after constructing RDF. Graph data are different from ordinary relational databases, which are nonrelational. At this time, the word sense disambiguation technology can be used to filter out the one that meets the requirements. Graph data perfectly solves the limitation that it is difficult to write for a large amount of data, and the computational efficiency of graph databases is more expressive and efficient compared with the model of a relational database. At present, Neo4j, Open Link, Big data, and other graph data are more popular, and the mainstream storage method of knowledge graph storage in the relational database is combined with graph database storage. In the Neo4j graph database, entities can be used not only for attributes but also for one or more labels at the nodes, in which the nodes represent the entities in the triad and their attributes; the edges represent the relationships in the knowledge graph triad, and the relationships not only have attributes but also have directional representation. The edges used to connect two nodes in the triad are directed edges and can represent the relationship between the entity and other entity and some additional attributes.

After the first two steps, many knowledge fragments can be obtained, and these knowledge fragments are a series of basic fact expressions [20]. As the last step of the knowledge graph construction process, in the knowledge processing stage, the ontology is firstly constructed so that the knowledge graph can have the concepts of upper and lower layers, and the knowledge fragments can form a structured and networked knowledge system to pave the way for the next knowledge representation reasoning using the knowledge graph. Because the quality of the new knowledge obtained through knowledge processing is uneven, to ensure the quality of the knowledge graph, the new knowledge obtained through knowledge processing needs to be evaluated and processed (part of which requires manual participation), and only the new knowledge that passes the evaluation can be included in the knowledge graph. At present, the models of information retrieval mainly include Boolean model, vector space model, and probability model. In the process of information retrieval, there are often multiple retrieval contents that meet the requirements.

**Figure 5:**
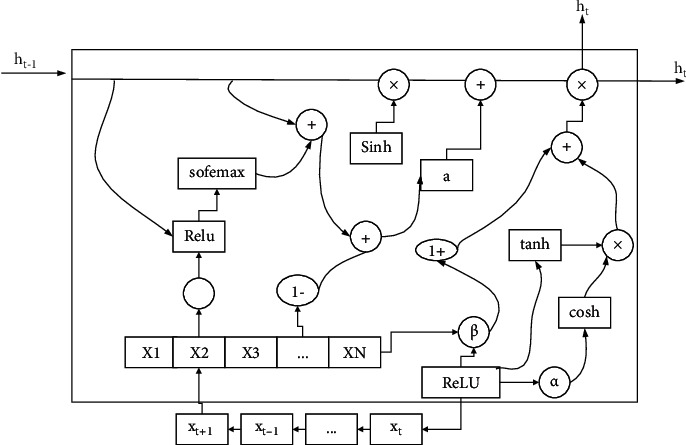
Schematic diagram of the graph construction algorithm flow.

The remotely supervised model combining syntactic dependency tree and ontology constraints first screens relational instances in the existing knowledge graph as training corpus and combines ontology corpus from ontology-related domains as constraints. The flowchart of the model algorithm is shown in Figure 5. Firstly, in the input layer, to address the problem of insufficient consideration of positional features in the input feature information, the model applies the positional weights of words in the sentence obtained from the syntactic dependency tree and combines them with the word vectors obtained through pretraining to obtain the input vectors. To address the problem that the final extracted relations do not conform to common sense, an ontology constraint layer of the ontology domain is added to the final output layer to constrain the extracted results through the ontology so that the extracted relations are more consistent with the domain common sense.

A reference for determining the construction details of HWE-IAN is provided; then, the BERT pretraining model is fused with the traditional word embedding, and the traditional word embedding is improved by using part of speech and position information. Interaction and mining of deeper feature correlation information to complete the construction of the entire HWE-IAN model are performed. The model starts with a feature engineering and extraction module, which includes a syntactic dependency tree, a word vector representation, and a position vector input layer. In the text input, words that are distant from words that possess relational attributes may have their weights assigned linearly lower because of the text representation. To address this problem, the model combines the word vector representation of all words in the input sentence with the syntactic dependency tree and the syntactic distance to generate the corresponding positional weights of each word in the sentence and then splices them with the positional vectors to obtain the final model input vector.

The positional features of words in the text are important for the relationship extraction task. Because the words of relationship attributes are close to the entities, that is, the closer the words are to the entities, the better they reflect the relationship between the entities. Therefore, before the feature extraction module, the two entities in the sentence are first marked and the relative distance between each word in the sentence and the two entities is calculated and transformed into a position vector, which is then spliced with the obtained word vector. In this way, the structured features of each word of the sentence can be obtained more accurately.

## 5. Neural Network Algorithm Performance Analysis

**Figure 6:**
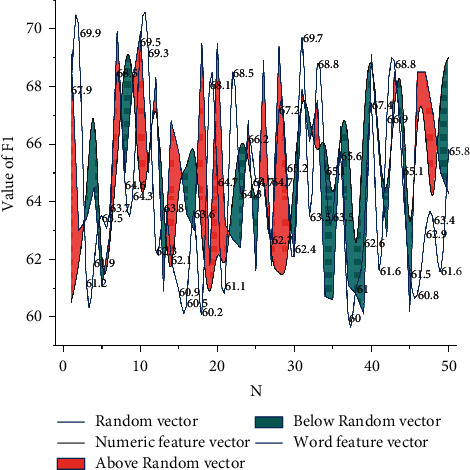
Comparison of the results of different feature word vectors.

When natural language is processed, the first step is to convert these linguistic symbols into numbers that can be processed by machines. The previous approach to text conversion was basically to represent each word in a sentence as a one-hot. However, as the number of texts increases dramatically, this approach consumes a lot of memory resources and cannot express any semantic information in the sentence, and words become completely independent of each other [21]. Word vectors are popular as an alternative way of text representation because they overcome the two problems proposed above. It is independent of the amount of data, can represent each word as a vector of fixed dimensionality, and can represent certain semantic information. In recent years, the literature on word vectors has emerged, and the quality of word vectors trained with different features, different corpora, and different models varies somewhat. In this paper, we discuss the impact of word vectors trained with different features on model results, comparing three types of vectors with random values, words as features, and Chinese characters as features.  Figure 6 shows the results of the comparison experiments. The vector with word context as the feature can get the largest F1 value and obtain the best result.

**Figure 7:**
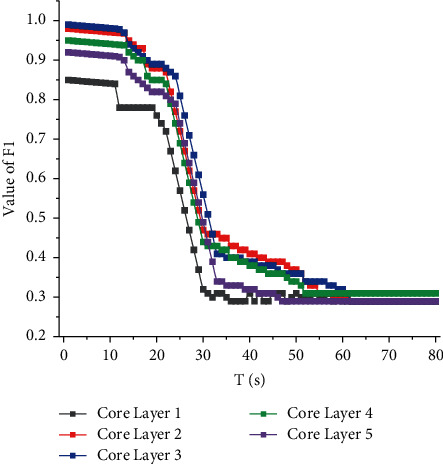
Comparison of results for different number of core layers.

This lexicon-driven method has the disadvantage of not being able to deal with the blurred word boundary well, and it cannot well identify words that do not exist in the lexicon. However, the effectiveness of the model is not positively correlated with the depth, so we did a series of comparison experiments to find the most suitable number of layers for our model. The experimental results are shown in Figure 7. The best results are obtained when the number of core layers is 0.3. This is probably because the amount of data in the training set is limited, and when the depth of the model increases, the complexity of the model increases greatly, causing it to learn a large amount of noise in the training set, which in turn causes the model to overfit.

Then, the study of methods related to semantic role annotation is described, and this section can be classified into two categories according to the processing methods used, namely, machine learning-based methods and deep learning-based methods. In general, machine learning-based methods use four steps to solve the task, namely, preprocessing, meta-identification, role annotation, and postprocessing. Related research has focused on the middle two parts. Usually, argument recognition can be treated as a binary classification task, and role labeling will be treated as a multiclassification task. The common idea is to construct feature templates of the argument elements to be classified and then select a classifier to process them. Therefore, much attention has been focused on how to construct better features and use better classifiers. The new generation of methods is also based on these two starting points for improvement. The difference is that using deep learning models as a framework allows merging recognition and labeling into the same part. In addition, the ability to extract rich features using neural networks and abandoning the reliance on manual construction of feature templates solve the problem of error accumulation and heavy resource consumption in traditional methods. When dealing with issues such as semantic analysis, knowledge graphs have the capabilities of logical reasoning, interpretability, natural correlation, efficient and transparent sharing, and visualization.

## 6. Analysis of the Results of Chinese Semantic Mapping Construction

After users input text information, the input text information is parsed based on the knowledge graph, which is the first step of knowledge inference Q&A in the whole knowledge representation system. When parsing the user's question and answer by the knowledge graph of web terms constructed in this paper, the accuracy of the analysis results of knowledge inference rules can be guaranteed by using the method of rule template design because the scope of knowledge inference questions and answers for web terms is relatively limited. Therefore, this paper selects the way of template rules to realize knowledge inference questions and answers in the knowledge representation system to semantically analyze the user's input text. After classifying the user's input text information by the utterance classification algorithm, the key entity information in the term input text information obtained by the named entity recognition algorithm is combined with the relationship extraction algorithm to obtain the relationship attributes between different entities, and the corresponding knowledge query template is used to obtain the inference results. The word sense disambiguation technology is introduced into the text classification problem. By judging the semantic category of the ambiguous words in the text, the category of the entire text can be judged, and then, the text data can be processed in large batches. Therefore, the word sense disambiguation technology is very important for text classification effect.

**Figure 8:**
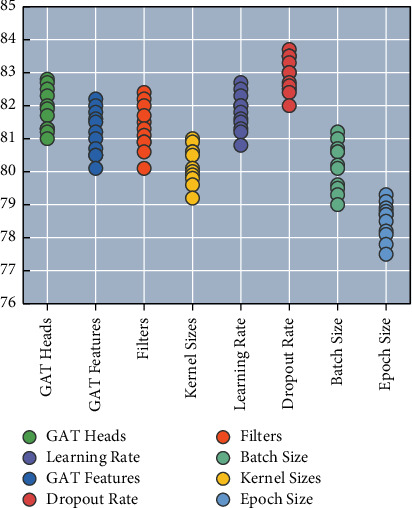
Chinese knowledge graph results.

Then comes the fusion with the data in the relational database: the process of aligning and fusing the data in the relational database and the microblog comment corpus is to solve the problem of inconsistent data formats and duplicate data in the knowledge that exists in the two kinds of data. The data fusion process starts with aligning and fusing the two kinds of data by adjusting the data in a uniform format and then using the traversal algorithm to clean the duplicated data and finally get the data that can be stored in the graph database. The data obtained after data fusion, and after obtaining the connections between knowledge according to the relationship extraction model, are stored in the Neo4j graph database used by the knowledge representation system in the form of a triad, and finally, the knowledge graph of network terms is obtained as shown in Figure 8.

**Figure 9:**
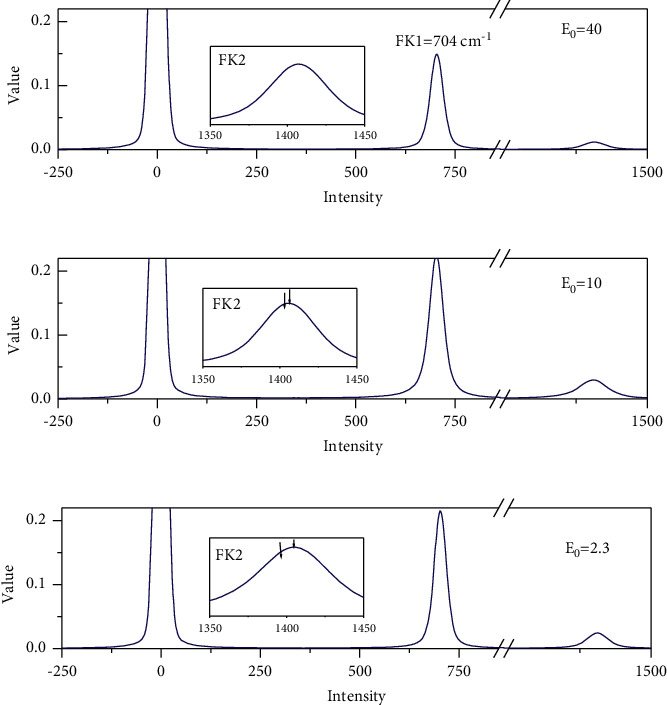
Hyperparameter results of the model.

A grid search is performed on the validation set for the hyperparameters, and the determined hyperparameters are shown in Figure 9. In the model for determining the poetry topics, the average pooling layer is used; the 2layer GAT is used to learn the node representation of the ancient poetry Atlas. In the model for analyzing the emotion of poems, CNN layers are used; 1layer GAT is used to learn the node representation of the ancient poetry Atlas. gat Heads indicate the number of heads in each layer of GAT, and GAT Features indicate the number of features output from each layer of GAT. Filters indicate the number of filters, and Kernel Sizes indicate the size of different convolutional kernels.

The reason for this phenomenon is that if we use the “random initialization word vector,” BERT can effectively capture the context information, including the n-gram information of the text and average the pool layer in the task of determining the theme of poetry. In the task of determining poetic themes, if we use “random initialized word vector + average pooling layer,” we cannot capture the connection between words, but by adding BERT, we add the contextual information of words, so adding BERT has great improvement. In the task of analyzing the emotion of poems, the original model is “random initialized word vector + CNN layer,” the CNN layer itself can capture the n-gram information of text, and then adding BERT, the contextual information contained in BERT may become noise and affect the effect of the model.

The effect of CNN-pretrain is 3.15% and 1.42% higher than CNN-rand on 2 this task, and the effect of GRU-pretrain is 3.91% and 5.09% higher than GRU-rand on this peal task, indicating that the initialization of word vectors is very important for model training, and pretraining word vectors using a large-scale corpus in the same domain as the training data and pretraining the word vectors with the same domain as the training data can significantly improve the model effect. In the task of determining poetry topics, GRU-rand and GRU-pretrain are 0.61% and 1.38% more effective than CNN-rand and CNN-pretrain, respectively; in the task of analyzing poetry emotions, CNN-rand and CNN-pretrain are 24.99% and 21.89% more effective than GRU-rand and GRU-pretrain, respectively. The results of pre training were improved by 25.97% and 22.13%, respectively. CNN and RNN have different preferences for different tasks and data, and RNN performs better on the task of determining poetry topics and CNN performs better on the task of analyzing poetry emotions.

## 7. Conclusion

In this paper, we propose a method to construct a Chinese knowledge map and use it to obtain a knowledge map of ancient poems with comprehensive content coverage, structured hierarchy, and containing semantic links of words. Using the ancient poetry map, poems can be better analyzed from various dimensions. Ancient poetry mapping is indispensable for data analysis of poetry, and it can effectively assist literary research from a semantic perspective. In addition, ancient poetry mapping can be applied to various inference and analysis tasks on ancient poetry, providing the necessary knowledge for these tasks, thus enabling a better understanding of poetry by machines. Named entity recognition (NER) is one of the key algorithms in building knowledge graphs, and named entity recognition algorithms in natural language processing toolkits rely heavily on the manual production of features and domain-specific knowledge to learn efficiently from the small, supervised training corpus available. The BERT-CNN network model utilized in this paper relies on two sources of information about words: character-based word representations learned from a supervised corpus, and unsupervised word representations learned from an unlabeled corpus, improving the accuracy of recognizing web-based term entities in text utterances. The experimental results show that semantic analysis of web phrases using knowledge graphs makes it possible to obtain the semantics of web phrases and interpret them more quickly and efficiently. This helps to understand the trend and thought trends of Internet users, improve their understanding and application of Internet phrases, and guide them to treat this language phenomenon more rationally.

## Data Availability

The data used to support the findings of this study are available from the corresponding author upon request.
